# ITGAV Promotes the Progression of Head and Neck Squamous Cell Carcinoma

**DOI:** 10.3390/curroncol31030099

**Published:** 2024-03-01

**Authors:** Lingyi Xu, Jeremy G Barrett, Jiayi Peng, Suk Li, Diana Messadi, Shen Hu

**Affiliations:** 1 School of Dentistry, University of California, Los Angeles, CA 90095, USA; lingyixu01@g.ucla.edu (L.X.); jer.barrett@gmail.com (J.G.B.); jypeng@g.ucla.edu (J.P.); dmessadi@dentistry.ucla.edu (D.M.); 2 Jonsson Comprehensive Cancer Center, University of California, Los Angeles, CA 90024, USA

**Keywords:** head and neck squamous cell carcinoma, integrin, integrin αV, SOX11

## Abstract

Head and neck squamous cell carcinoma (HNSCC) refers to the malignancy of squamous cells in the head and neck region. Ranked as the seventh most common cancer worldwide, HNSCC has a very low survival rate, highlighting the importance of finding therapeutic targets for the disease. Integrins are cell surface receptors that play a crucial role in mediating cellular interactions with the extracellular matrix (ECM). Within this protein family, Integrin αV (ITGAV) has received attention for its important functional role in cancer progression. In this study, we first demonstrated the upregulation of *ITGAV* expression in HNSCC, with higher *ITGAV* expression levels correlating with significantly lower overall survival, based on TCGA (the Cancer Genome Atlas) and GEO datasets. Subsequent in vitro analyses revealed an overexpression of ITGAV in highly invasive HNSCC cell lines UM1 and UMSCC-5 in comparison to low invasive HNSCC cell lines UM2 and UMSCC-6. In addition, knockdown of ITGAV significantly inhibited the migration, invasion, viability, and colony formation of HNSCC cells. In addition, chromatin immunoprecipitation (ChIP) assays indicated that SOX11 bound to the promoter of ITGAV gene, and SOX11 knockdown resulted in decreased ITGAV expression in HNSCC cells. In conclusion, our studies suggest that ITGAV promotes the progression of HNSCC cells and may be regulated by SOX11 in HNSCC cells.

## 1. Introduction

Head and neck squamous cell carcinoma (HNSCC) originates from the squamous cells in the head and neck region, which include the oral cavity, pharynx, nasal cavity, paranasal sinuses, and salivary glands. It ranks as the seventh most common cancer in the world, with high prevalence in South and Southeast Asia [1,2]. The high-risk population for HNSCC includes individuals with a prolonged history of smoking and alcohol consumption. Moreover, there is a rise in the incidence of oropharyngeal cancer among younger populations, which is linked to human papillomavirus (HPV) exposure [3]. The current treatment approaches, such as surgical resection, radiation therapy, and chemotherapy, are largely incurable for HNSCC and significantly affect the overall quality of life of the patients [4]. Hence, it is important to develop new therapeutic treatment of HNSCC.

It is well known that extracellular matrix (ECM) genes are critical for cellular survival signals and involved in the initiation of cancer invasion and metastasis [5]. ECM proteins may transduce the signals through transmembrane proteins involved in promoting cell survival, migration, proliferation, and differentiation. Integrins are a widely expressed family of cell surface membrane receptors that attach the cell to the extracellular membrane, mediate cell–cell interactions and promote outside-in and inside-out signaling in ligated and unligated forms [6,7,8,9,10]. They are heterodimers composed of non-covalently linked α and β subunits. Each subunit has a substantial extracellular domain, a singular membrane-spanning domain, and a short, non-catalytic cytoplasmic tail [11]. In vertebrates, 18 α and 8 β subunits have been identified, which can combine to form 24 distinct integrin receptors [12]. Specifically, ITGAV, a subunit of the integrin receptor, can pair with β1, β3, β5, β6, or β8 subunits [13]. Integrins are categorized based on their binding specificities, which include receptors for Arg-Gly-Asp (RGD) peptide motifs, collagen, laminin, and leukocyte-specific integrins [14]. Among these integrin receptors, several exhibit ligand specificities. For instance, integrin α5β1 functions as a receptor specifically for fibronectin, whereas integrin α5β3 demonstrates an affinity for a broad spectrum of fibrous proteins, including fibronectin, fibrinogen, and vitronectin [7]. The integrins α5β1, α5β3, and α5β5, all incorporating the ITGAV subunit, are implicated in regulating angiogenesis through distinct pathways [15]. Notably, overexpression of α5β3 has been correlated with metastasis and increased tumor aggressiveness in melanoma, breast, and pancreatic cancers [7,16,17,18]. Furthermore, enhanced expression of ITGAV in its dimerized form as αvβ3 plays a pivotal role in the transendothelial migration of several invasive human cancers [19]. Integrin αvβ3, initially identified in association with angiogenesis, is uniquely expressed in angiogenic endothelial cells, contrasting its absence in normal endothelial cells [20,21]. Moreover, elevated expression of ITGAV has been documented in various solid tumors, including those of the bladder, colorectum, prostate, and breast [13,15,22,23]. However, the specific role of ITGAV in head and neck cancers remains to be elucidated.

SOX11 (SRY-Box 11), a transcription factor involved in cell fate determination, plays a vital role during embryogenesis [24]. The SOX gene family is characterized by the high mobility group (HMG) domain initially identified in the sex-determining gene SRY on the Y chromosome. SOX11, located on chromosome 2p25.2, is part of the SOXC group of transcription factors, along with SOX4 and SOX12 in most vertebrates [25]. Abnormal upregulation of SOX11 has been reported in various cancers; however, the prognostic implications of SOX11 expression appear to be contingent on the specific cancer type [26]. In our recent study, the expression of ITGAV was found to be downregulated in HNSCC cells with SOX11 knockdown [27]. Therefore, the objective of our study is to investigate the role of ITGAV in HNSCC cells, focusing on highly invasive cell lines UM1 and UMSCC-5. Additionally, we aim to explore SOX11 as a potential upstream regulator for ITGAV. 

## 2. Materials and Methods

### 2.1. Analysis of ITGAV Gene Expression in HNSCC and Its Clinical Significance

The publicly available gene expression data from the Gene Expression Omnibus (GEO) (https://www.ncbi.nlm.nih.gov/gds/, accessed on 10 October 2023) were utilized to evaluate the expression level of *ITGAV* in HNSCC and normal tissues. Specifically, the GEO series with the accession numbers GSE6631, GSE13601, and GSE30784 were employed. Additionally, publicly available genomic data from The Cancer Genome Atlas (TCGA), hosted on the NCI Genomic Data Commons (GDC, data release 38.0) Data Portal (https://portal.gdc.cancer.gov/, accessed on 10 October 2023), were also used. The R program (version 4.1.3) was used to organize/analyze the gene expression data, and mRNA expression levels were log2-transformed. The survival analysis was performed using TCGA data and the R program was used to generate survival plots.

### 2.2. Cell Culture

Four HNSCC cell lines UM1, UM2, UMSCC-5, and UMSCC-6 obtained from Dr. Yong Kim at UCLA School of Dentistry were used in this study. These cell lines were cultured in Dulbecco’s Modified Eagle Medium (DMEM) (Invitrogen Life Technologies, Carlsbad, CA, USA), supplemented with 10% fetal bovine serum (Gemini Bio Products, West Sacramento, CA, USA), and an antibiotic-antimycotic solution containing 100 U/mL penicillin and 100 μg/mL streptomycin (1%) (Invitrogen Life Technologies, Carlsbad, CA, USA). The cells were maintained at 37 °C, 5% CO_2_ in a humidified incubator and passaged when the confluency reached 80–90%.

### 2.3. Quantitative Polymerase Chain Reaction (qPCR)

UM1, UM2, UMSCC-5, and UMSCC-6 cell lines were cultured to 80–90% confluency in 6-well plates in preparation for mRNA extraction. For each plate, 400 μL of RB buffer was applied for 10 min, followed by collection of the cell lysate. The lysate was then homogenized and subjected to centrifugation at 1500 rpm for 5 min. The supernatant obtained was processed using the RNAEasy kit (Genesee Scientific, Morrisville, NC, USA) to isolate the mRNA. Subsequent to mRNA extraction, it was reverse transcribed into cDNA using the Superscriptase III kit (Invitrogen Life Technologies, Carlsbad, CA, USA), and its concentration was quantified with the Nanodrop spectrophotometer. For qPCR, 1 μL of the cDNA sample (diluted 1:10) was added to a reaction mixture in a 50 μL microcentrifuge tube. This mixture included 10 μL of SYBR Green Supermix 2×, 0.2 μL of each forward and reverse primer (10 μM), and sufficient sterilized water to bring the total volume to 20 μL. The qPCR conditions were as follows: denaturation at 94 °C for 40 s, annealing at 55 °C for 30 s, extension at 68 °C for 90 s across 40 cycles, and a final extension phase at 68 °C for 8 min. The resulting C_q_ values were recorded and analyzed to determine fold changes in gene expression using the ΔΔCT method. Details of the *ITGAV* primers employed are provided in Table 1.

### 2.4. Western Blotting

Cell lysates were prepared, and proteins were extracted using the 2-D gel rehydration buffer. The total protein concentration was determined using the Bradford assay. Proteins in quantities ranging from 15 μg to 20 μg were denatured and subsequently loaded into the wells of polyacrylamide gels (4–12%), prepared according to the manufacturer’s guidelines. Protein separation was conducted using the MiniPROTEAN^®^ Tetra Vertical Electrophoresis Cell (Bio-Rad, Brea, CA, USA). Following this, the proteins were transferred onto nitrocellulose membranes (Santa Cruz Biotechnology, Santa Cruz, CA, USA) with the Trans-Blot SD semi-dry transfer cell (Bio-Rad, Brea, CA, USA). The membranes were blocked with 5% non-fat dry milk in TBST for one hour at ambient temperature and then incubated overnight at 4 °C with primary antibodies against ITGAV, SOX-11, and GAPDH (Santa Cruz Biotechnology, Dallas, TX, USA). The membranes were then washed thrice with TBST, followed by an hour-long incubation at room temperature with an appropriate secondary antibody (Santa Cruz Biotechnology, CA, USA). Three additional washes with TBST were performed, and then, signal detection was performed using the ECL-Plus Western Blotting reagent kit (GE Healthcare, Little Chalfont, UK). The films were developed in a dark room, scanned, and analyzed using the NIH ImageJ Software (Version 1.53).

### 2.5. siRNA Knockdown of ITGAV in UM1 and UMSCC5 Cells

UM1 and UMSCC-5 were maintained in 6-well plates. When the confluency reached 70%, cells were transfected with the mixture of siRNA and lipofectamine RNAiMAX transfection reagent (Invitrogen, Carlsbad, CA, USA) at a ratio of 30 pmol of siRNA to 9 μL RNAiMAX per well. After incubating for 48 h, the siRNA knockdown was stopped, and the cells with ITGAV knocked-down were used for later experiments. 

### 2.6. Transwell Migration Assay

The migration capability of cancer cells was assessed using the Transwell (Corning^TM^3422, Corning, NY, USA). Both the controlled and siRNA treated cells were resuspended in serum-free medium and loaded into Transwell inserts at a density of 1 × 10^5^ cells/well. Each lower chamber was loaded with 500 μL of complete medium. After 48 h, the migrated cells were fixed with the Hema 3^TM^ Stat Pack (Fisher Scientific, Pittsburgh, PA, USA). Three areas of the migrated cells were selected randomly and counted. The average number of migrated cells was calculated using Image J (Version 1.53).

### 2.7. Transwell Invasion Assay

The invasive capacity of cancer cells was assessed using the Matrigel-coated Transwell chambers (Corning^TM^354480, Corning, NY, USA). Both the control and siRNA treated cells were resuspended in serum-free medium and loaded into the Transwell inserts at the density of 1 × 10^5^ cells/well, while the lower chambers were loaded with 500 μL complete media. The invaded cells were stained and counted the same way as in the migration assay after 72 h. 

### 2.8. Colony Formation Assay

Complete media were loaded into 6-well plates. The control and siRNA transfected cells were plated in each well at a density of 1000 (UMSCC-5) or 3000 (UM1) cells/well. The cells were cultured at 37 °C, 5% CO_2_ in a humidified incubator for 14 days (UM1) and 28 days (UMSCC-5), respectively, before they were fixed with 3.7% formaldehyde and stained with 0.1% crystal violet. The cell images were captured, and the average number of cells in each group was acquired with the ImageJ software (Version 1.53).

### 2.9. CCK-8 Assay

Cells were resuspended in the complete media and seeded in the 96-well plates at a density of 5000 cells per well. Following a 24 h incubation, the cells were transfected with siRNA at the same concentration as above. Subsequently, 10 μL of CCK-8 reagent (Sigma-Aldrich, St. Louis, MO, USA) was added to each well, and the plates were returned to the incubator. Two hours later, the optical density (OD) at 450 nm was measured using a plate reader (BioTek Instrument Inc., Winooski, VT, USA). This procedure was replicated at 24 h intervals, with readings taken at 24, 48, 72, and 96 h post-transfection. The mean OD values were calculated and plotted.

### 2.10. Chromatin Immunoprecipitation (ChIP)

The ChIP assays were performed on UM1 and UMSCC-5 cells utilizing the ChIP assay kit (Millipore, Billerica, MA, USA), according to the manufacturer’s protocol. After PBS wash, the cancer cells were cross-linked with 1% formaldehyde on a Petri dish for 10 min at room temperature, followed by quenching with 125 mM glycine. Subsequent to two washes with cold PBS, the cells were harvested in RIPA buffer (150 mM NaCl, 1% Igepal CA-630, 0.5% deoxycholate, 0.1% SDS, and 50 mM Tris-HCl at pH 8) and sonicated to yield DNA fragments ranging from 200 to 1000 base pairs in size. For the immunoprecipitation process, 1 mg of protein extract underwent pre-clearance with 30 μL of Protein A Agarose/Salmon Sperm DNA (50% Slurry) for 30 min. After centrifugation to remove the agarose, the supernatant was incubated with 5 μg of anti-SOX11 antibody (Santa Cruz Biotechnology) overnight at 4 °C, followed by an additional hour-long incubation at 4 °C with 30 μL of Protein A Agarose/Salmon Sperm DNA (50% Slurry). Subsequent washes included low-salt, high-salt, and LiCl immune complex wash buffers, as well as TE buffer. The immunocomplexes were then collected and eluted with 1% SDS/0.1 M NaHCO_3_ for 10 min at 65 °C. The reversal of histone-DNA crosslinks was achieved by treatment with 200 mM NaCl at 65 °C for 4 h. The final step involved purifying the DNA fragments using the DNA Clean/Concentrator kit (Zymo Research, Irvine, CA, USA), which then served as templates for qPCR reactions, using the primers specific to the ITGAV gene promoter (Table 1).

### 2.11. Statistical Analysis

All experimental procedures were replicated in triplicate, except where explicitly stated otherwise. The data were expressed as mean ± standard deviation (SD). Statistical analysis was carried out using either independent samples *t*-test or analysis of variance (ANOVA), as appropriate. The data were plotted using the GraphPad Prism (version 7.0, GraphPad Software Inc., San Diego, CA, USA). *p*-values less than 0.05 were considered statistically significant.

## 3. Results

### 3.1. Association of ITGAV Overexpression with Poor Prognosis in HNSCC 

**Figure 1:**
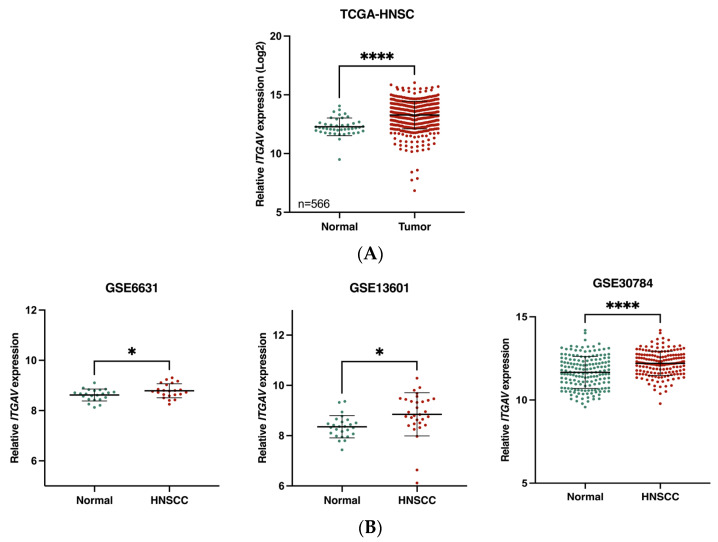
Overexpression of ITGAV gene in HNSCC. (**A**) According to TCGA datasets, ITGAV gene expression was upregulated in HNSCC (**** *p* < 0.0001). (**B**) According to the GSE datasets, the expression level of ITGAV gene was significantly higher in HNSCC tissues compared to normal adjacent tissues (* *p* < 0.05, **** *p* < 0.0001).

**Figure 2:**
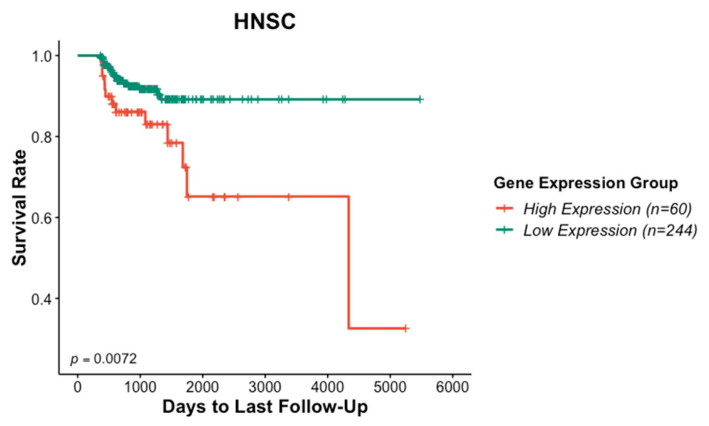
Patients’ survival rates of HNSCC patients with high and low ITGAV gene expression. HNSCC patients with high levels of ITGAV gene expressions exhibited worse survival than the patients with low ITGAV gene expression.

Analysis of TCGA datasets revealed a significant overexpression of the ITGAV gene in HNSCC (Figure 1A). Additionally, ITGAV gene expression was significantly upregulated in HNSCC tumor versus adjacent normal tissues according to three GEO datasets (accession numbers GSE6631, GSE13601, and GSE30784) (Figure 1B). Patients with HNSCC exhibiting higher ITGAV gene expression demonstrated worse survival outcomes compared to those with lower expression levels (Figure 2).

### 3.2. ITGAV Expression in High- and Low-Invasive HNSCC Cell Lines 

**Figure 3:**
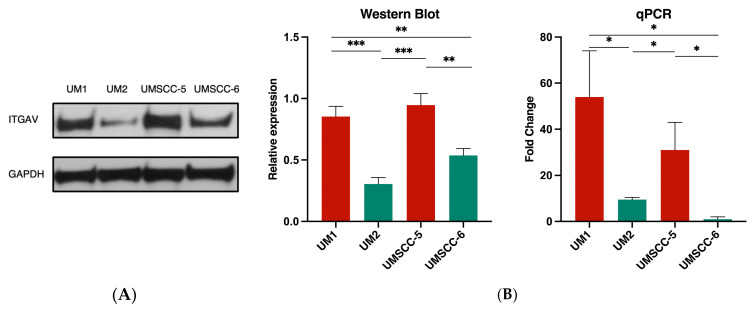
ITGAV gene and protein expression in high- and low-invasive HNSCC cell lines. (**A**) Western blotting analysis of the differential ITGAV protein expression level in high-invasive cancer cell lines (UM1 and UMSCC-5) and low-invasive cancer cell lines (UM2 and UMSCC-6). Quantification of Western blotting shows that the expression levels of ITGAV in UM1 and UMSCC-5 cells are significantly higher than in UM2 and UM6 cells (* *p* < 0.05, ** *p* < 0.001, *** *p* < 0.001). (**B**) qPCR analysis of *ITGAV* expression in high-invasive cancer cell lines (UM1 and UMSCC-5) and low-invasive cancer cell lines (UM2 and UMSCC-6). The result indicates a higher expression of *ITGAV* in highly invasive cancer cell lines (UM1 and UMSCC-5) compared to low-invasive cancer cell lines (UM2 and UMSCC-6) (* *p* < 0.05).

Western blot analysis demonstrated a significantly higher ITGAV expression in high-invasive cell lines UM1 and UMSCC5 in comparison to low-invasive cell lines UM2 and UMSCC-6 (** *p* < 0.01, *** *p* < 0.001) (Figure 3A). qPCR analysis also indicated a significant increase in ITGAV gene expression in UM1 and UMSCC-5 cells compared to UM2 and UMSCC-6 cells (Figure 3B). UM1 had a 44.5-fold increase over UM2 (* *p* < 0.05) and a 53-fold increase over UMSCC-6 (* *p* < 0.05), while UMSCC-5 had a 21.5-fold increase over UM2 (* *p* < 0.05) and 30-fold increase over UMSCC-6 (* *p* < 0.05). 

### 3.3. Knockdown of ITGAV in UM1 and UMSCC-5 Cells Inhibits Cell Migration, Invasion, Viability, and Proliferation 

**Figure 4:**
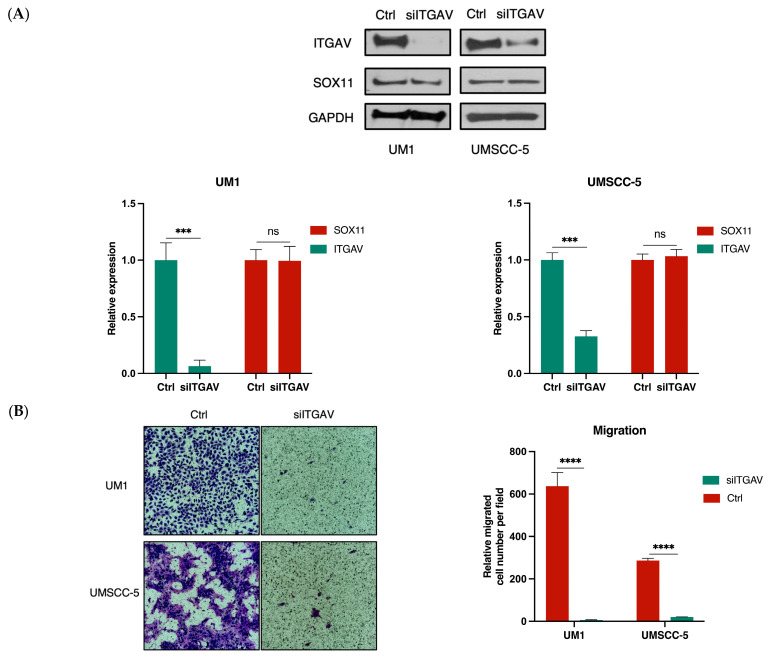

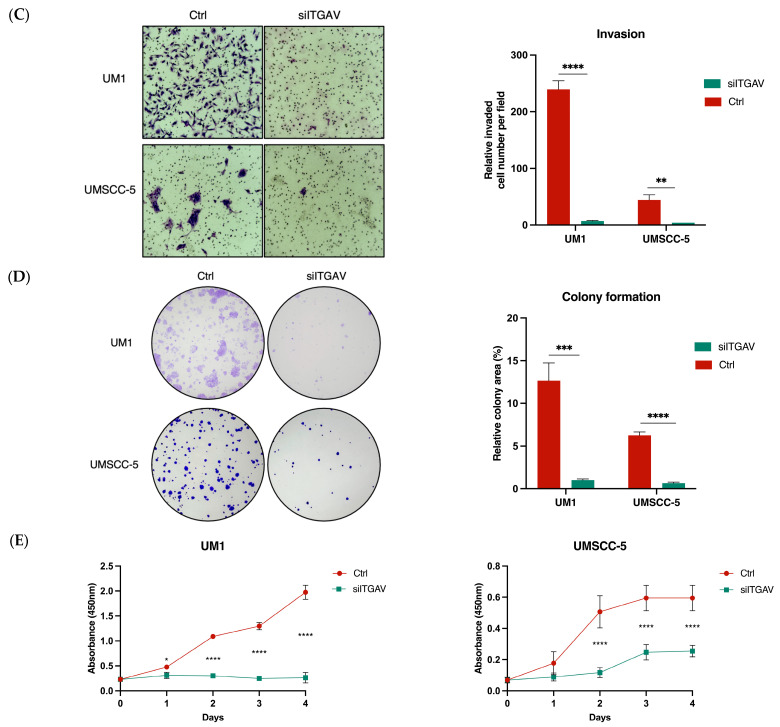
Knockdown of ITGAV in UM1 and UMSCC-5 cell lines inhibits cell migration, invasion, and proliferation. (**A**) ITGAV knockdown in both cell lines was confirmed by Western blotting. Significant downregulation of ITGAV protein expression after siITGAV transfection was observed in both cell lines. However, the expression level of SOX-11 after siITGAV knockdown was not significantly altered in both cell lines (ns stands for not significant, *** *p* < 0.001). (**B**) Knockdown of ITGAV significantly inhibits the migration of both UM1 and UMSCC-5 cells (**** *p* <0.0001). (**C**) Knockdown of ITGAV significantly inhibits the invasion of both UM1 and UMSCC-5 cells (** *p* < 0.01,**** *p* < 0.0001). (**D**) ITGAV knockdown significantly suppresses the ability of both UM1 and UMSCC-5 cells to form colonies (*** *p* < 0.001, **** *p* < 0.0001). (**E**) The CCK-8 assays indicate that ITGAV knockdown suppresses the proliferation of both UM1 and UMSCC-5 cells (* *p* < 0.05, **** *p* < 0.0001).

Western blot analysis confirmed that ITGAV protein expression was significantly lowered after being transfected with siITGAV (*** *p* < 0.001) (Figure 4A). Transwell migration assays were performed to analyze UM1 and UMSCC-5 cells’ migration capacity after siRNA knockdown. The results indicated that both UM1 and UMSCC-5 cells with siITGAV transfection showed significantly reduced migration capacity compared to their control groups, respectively (**** *p* < 0.0001) (Figure 4B). Similarly, the invasion capabilities of UM1 and UMSCC-5 cells after siITGAV transfection were investigated using the Matrigel invasion assay. As indicated in Figure 4C, both UM1 and UMSCC-5 cell lines exhibited a significant reduction in invasion capabilities (** *p* < 0.01, **** *p* < 0.0001). Knockdown of ITGAV in both HNSCC cell lines also inhibited cell proliferation (Figure 4D,E). The CCK-8 assay indicated a significant reduction in the viability of UM1 cancer cells transfected with siITGAV. This decrease was evidenced by the absorbance values measured at 450 nm for each group on successive days (* *p* < 0.05, **** *p* < 0.0001). UMSCC-5 cells exhibited a similar significant reduction in cell viability, except for the first day (**** *p* < 0.0001). Additionally, the colony formation assay indicated a significant decrease in the ability of both cell lines to form colonies after ITGAV knockdown (*** *p* < 0.001, **** *p* < 0.0001).

### 3.4. SOX11 Regulates ITGAV Expression in HNSCC Cells

**Figure 5:**
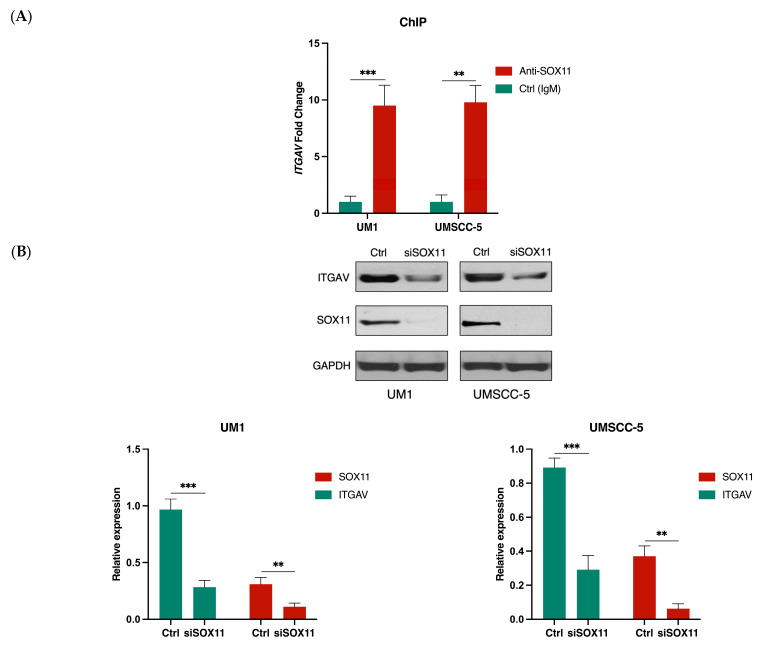
SOX11 regulates ITGAV expression in HNSCC cells. (**A**) ChIP-qPCR analysis of anti-SOX11- or IgG-immunoprecipitated DNA fragments from UM1 or UMSCC-5 cells. The result indicates a significantly higher enrichment of promoter fragments of ITGAV gene in both UM1 and UMSCC-5 cells by SOX11 (** *p* < 0.001, *** *p* < 0.001). (**B**) SOX11 knockdown downregulates the expression of ITGAV in HNSCC cells as revealed by Western blotting.

Since our previous studies suggested that ITGAV expression was downregulated in HNSCC cells with siSOX11 knockdown [27], a ChIP assay was performed to investigate if SOX11 binds to the promoter of ITGAV gene in UM1 and UMSCC-5 cells. qPCR was used to assess the DNA enrichment within the anti-SOX11 immunoprecipitated samples versus the IgG antibody immunoprecipitated samples (negative control). The results revealed a significantly higher enrichment of the fragments of ITGAV gene promoter in the anti-SOX11-immunoprecipitated samples compared to the IgG-immunoprecipitated samples (both UM1 and UMSCC-5 cell lines) (Figure 5A). Additionally, Western Blot analysis indicated that the knockdown of SOX11 led to a significant decrease in the expression levels of both SOX11 and ITGAV in both UM1 and UMSCC-5 cell lines (Figure 5B).

## 4. Discussion

HNSCC is one of the most common cancer types, ranking seventh in global incidence rates among all human cancers [1]. Many patients who undergo surgical removal of HNSCC experience tumor recurrence and/or develop a second primary HNSCC [28,29,30]. As concurrent chemoradiotherapy regimens are the mainstay of treatment for locally advanced HNSCC, challenges have emerged in the treatment of distant metastatic cancer [3]. These patients with metastatic diseases do not have an effective treatment option, and often the treatment has a significant impact on the quality of their life [4]. Nevertheless, the 5-year survival rate for HNSCC has barely improved over the past three decades [31]. The limited therapeutic options available for recurrent or metastatic HNSCC further contribute to its unfavorable prognosis [32]. Despite considerable progress in understanding this disease in recent decades, these achievements have not resulted in clinically meaningful breakthroughs [4]. Hence, the identification of new targets for therapeutic intervention is imperative to improve treatment outcomes.

In this study, analysis of both TCGA and GEO datasets revealed a significantly upregulated gene expression of ITGAV in HNSCC and various cancers, such as cholangiocarcinoma (CHOL), liver hepatocellular carcinoma (LIHC), lung squamous cell carcinoma (LUSC), and stomach adenocarcinoma (STAD) (Figure S1). Furthermore, survival analysis of the TCGA datasets revealed that HNSCC patients with higher ITGAV gene expression had a significantly worse overall survival rate compared to those with lower ITGAV expression (Figure 2). Analogous patterns were observed in patients with different types of cancer, wherein elevated expression of the ITGAV gene exhibited a notable correlation with diminished long-term overall survival rates across various cancer types, including bladder urothelial carcinoma (BLCA), colon adenocarcinoma (COAD), esophageal carcinoma (ESCA), liver hepatocellular carcinoma (LIHC), pancreatic adenocarcinoma (PAAD), sarcoma (SARC), and stomach adenocarcinoma (STAD) (Figure S2). This finding suggests that ITGAV may be a viable prognostic biomarker for HNSCC. Previous studies also indicated that significant upregulation of ITGAV correlated with the metastatic potential of cancer cells. For instance, inducing the expression of ITGAV [33] in a melanoma cell line was found to be associated with increased metastatic potential. Given the established relationship between ITGAV expression and survival outcomes, ITGAV may serve as a potential therapeutic target in HNSCC.

To elucidate the functional role of ITGAV in HNSCC, we first evaluated the expression of ITGAV between high-invasive UM1 and UMSCC-5 cell lines and low-invasive UM2 and UMSCC-6 cell lines. Our results clearly showed that ITGAV was significantly upregulated in highly invasive HNSCC cell lines, UM1 and UMSCC-5. Therefore, UM1 and UMSCC-5 cell lines were selected for further knockdown studies. Previous studies suggest that integrins may play a role in ECM remodeling by activating ECM protein degradation pathways. For instance, MMP-2 and MMP-9 are enzymes that targets type IV collagen, a key ECM protein. Integrin αvβ3 is present in the multiprotein MMP-activating complex in cancer cells, activating MMPs and facilitating directed cellular invasion [11]. As we expected, knockdown of ITGAV expression significantly suppressed the capability for migration and invasion in both UM1 and UMSCC-5 cell lines. In addition, both CCK-8 and colony formation assays demonstrated that ITGAV downregulation significantly inhibited the proliferation of both UM1 and UMSCC-5 cancer cells. These results suggest that targeted intervention to suppress ITGAV expression may serve as an efficacious approach to impede HNSCC proliferation. Although ITGAV-targeting anti-cancer treatments have not been reported in clinical trials, new therapeutic agents targeting αv-containing integrins are currently being tested in clinical trials for fibrotic diseases such as idiopathic pulmonary fibrosis (IPF) and nonalcoholic steatohepatitis (NASH) [34,35].

Since ITGAV expression was found to be downregulated in two HNSCC cell lines following SOX11 knockdown [27], we further investigated if SOX11, a transcription factor, may serve as an upstream regulator of ITGAV. All SOX proteins predominantly bind to the conserved DNA motif TTGT [36], with slight variations in adjacent bases [36,37] influencing DNA binding efficiency, as mediated by the SOX-HMG signature motif [38]. Our ChIP assays revealed that SOX11 bound to the ITGAV gene promoter in UM1 and UMSCC-5 cells. In addition, based on the Western blot analysis (Figure 4A and Figure 5B), ITGAV knockdown did not affect SOX11 expression, whereas SOX11 knockdown led to significantly decreased ITGAV expression in HNSCC cells. Collectively, these findings indicate that SOX11 may act as an upstream regulator modulating ITGAV expression in HNSCC cells. 

In conclusion, our studies suggest that ITGAV contributes to the progression of HNSCC and may well serve as a prognostic biomarker in HNSCC. Since knockdown of ITGAV significantly impairs the proliferation of HNSCC cells, the employment of siRNA technology represents a viable therapeutic avenue and an alternative treatment strategy for HNSCC. Further investigation is imperative to delineate the regulatory role of SOX11 in the modulation of ITGAV expression in HNSCC. Nevertheless, there are two limitations of this study. First, the functional role of ITGAV was primarily investigated with HPV-negative HNSCC cells. HPV-positive HNSCC cells may be included for a comparative analysis. Second, animal model studies are needed to further confirm the role of ITGAV for promoting HNSCC invasion and metastasis in vivo. 

## Figures and Tables

**Table 1:** List of primers of *ITGAV* used for qPCR.

Gene	Forward Primer	Reverse Primer
*ITGAV* Promoter 1	TGCCCTGCGAATCCTTTCTT	CGTGTTTCTGCTGCTTAGCC
*ITGAV* Promoter 2	GCCTTATTTCACCGGTGTGC	AAGGATTCGCAGGGCAAAGA

## Data Availability

The data presented in this study are available upon request.

## Supplementary Materials

The following supporting information can be downloaded at: https://www.mdpi.com/article/10.3390/curroncol31030099/s1, Figure S1: Overexpression of ITGAV gene in various cancers; Figure S2: Patients’ survival rates of various cancer patients with high and low ITGAV gene expression.
