# Supplementary figures and images for: ITGAV Promotes the Progression of Head and Neck Squamous Cell Carcinoma
Source: Curr Oncol. 2024 Mar 1;31(3):1311–22. doi: 10.3390/curroncol31030099 (PMC10969037; doi:10.3390/curroncol31030099)

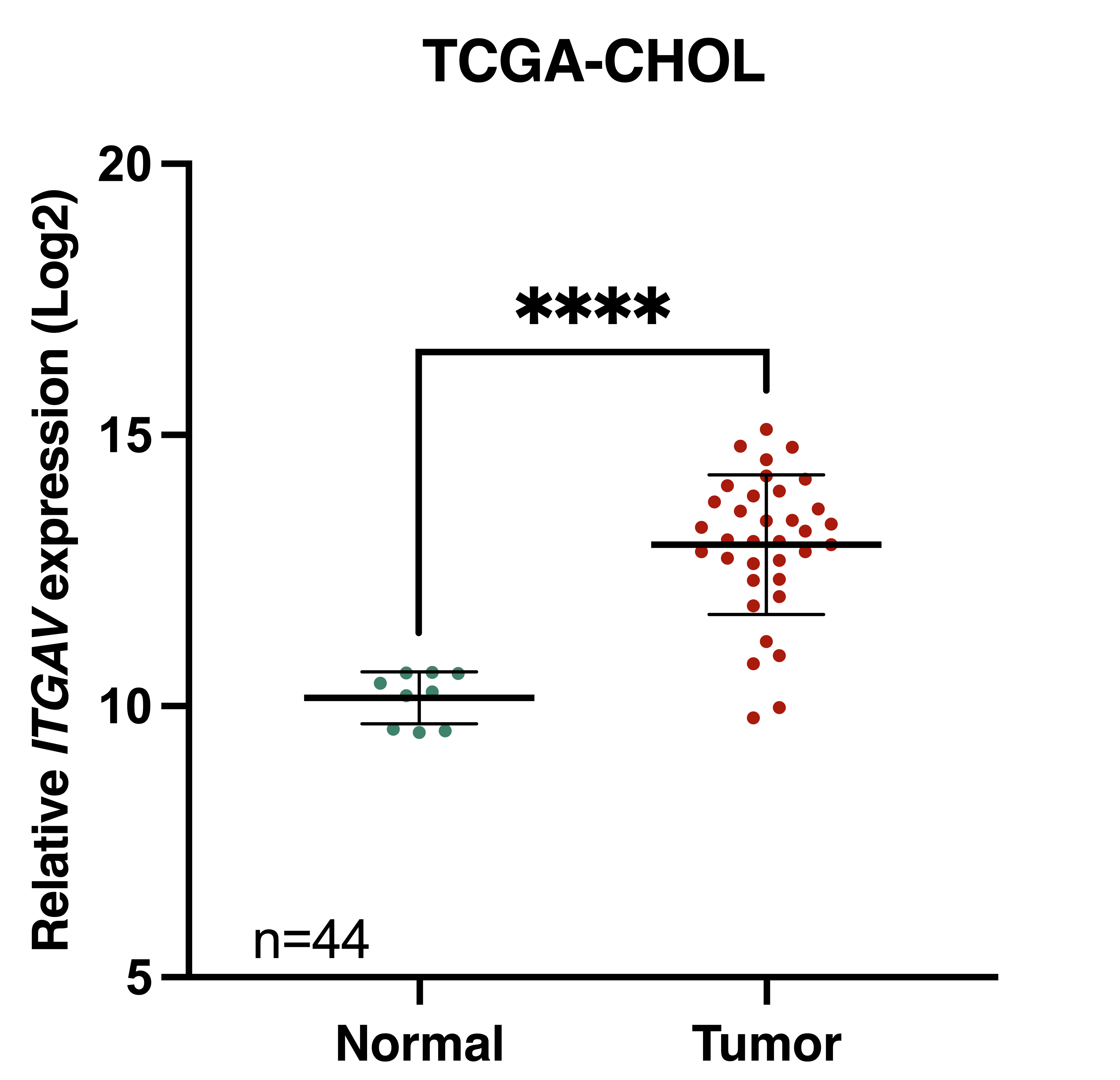

Supplement: Supplementary file 1 [file curroncol-31-00099-s001.zip › supplemental figures/Figure S1/CHOL.tiff]

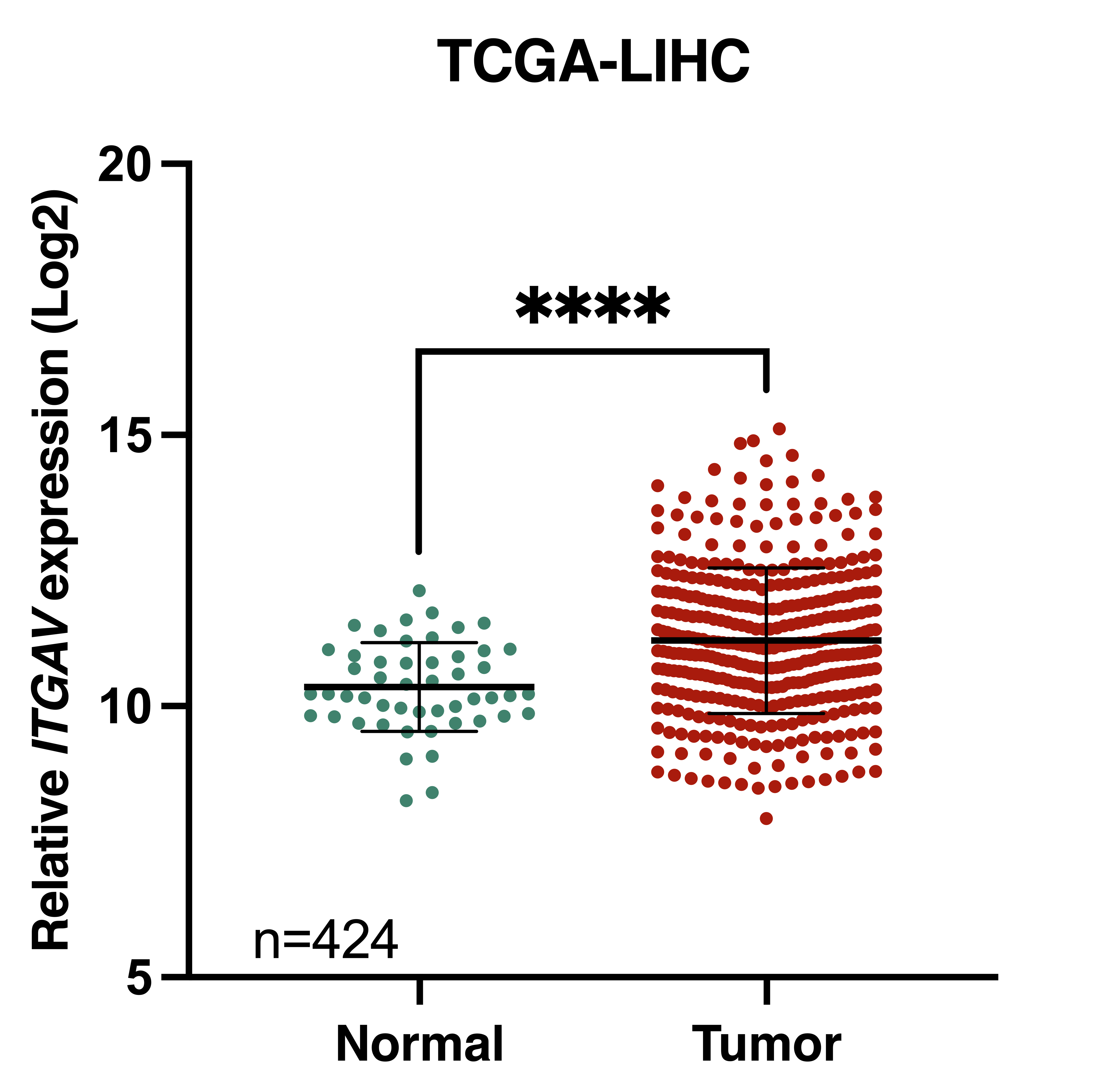

Supplement: Supplementary file 1 [file curroncol-31-00099-s001.zip › supplemental figures/Figure S1/LIHC.tiff]

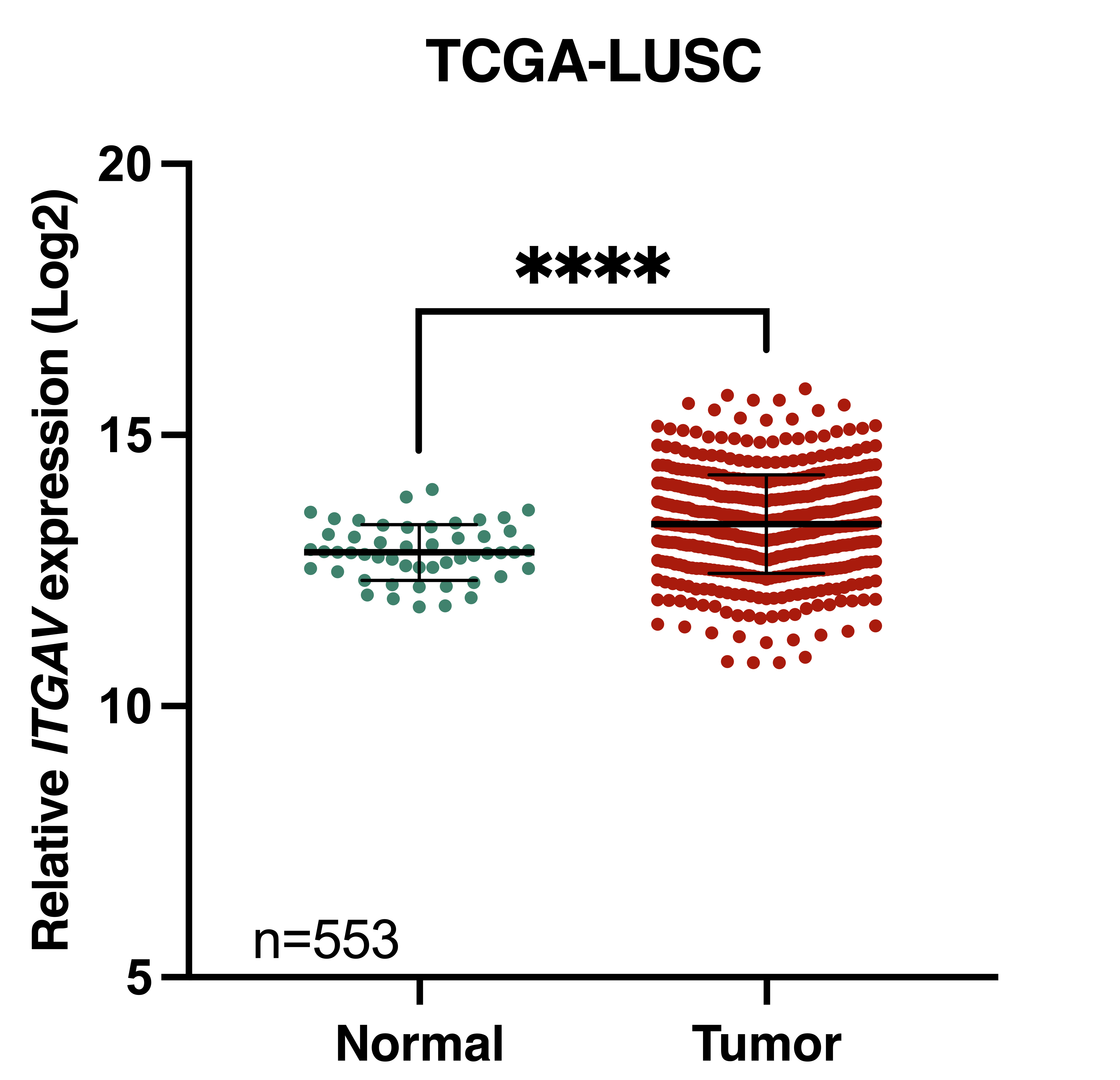

Supplement: Supplementary file 1 [file curroncol-31-00099-s001.zip › supplemental figures/Figure S1/LUSC.tiff]

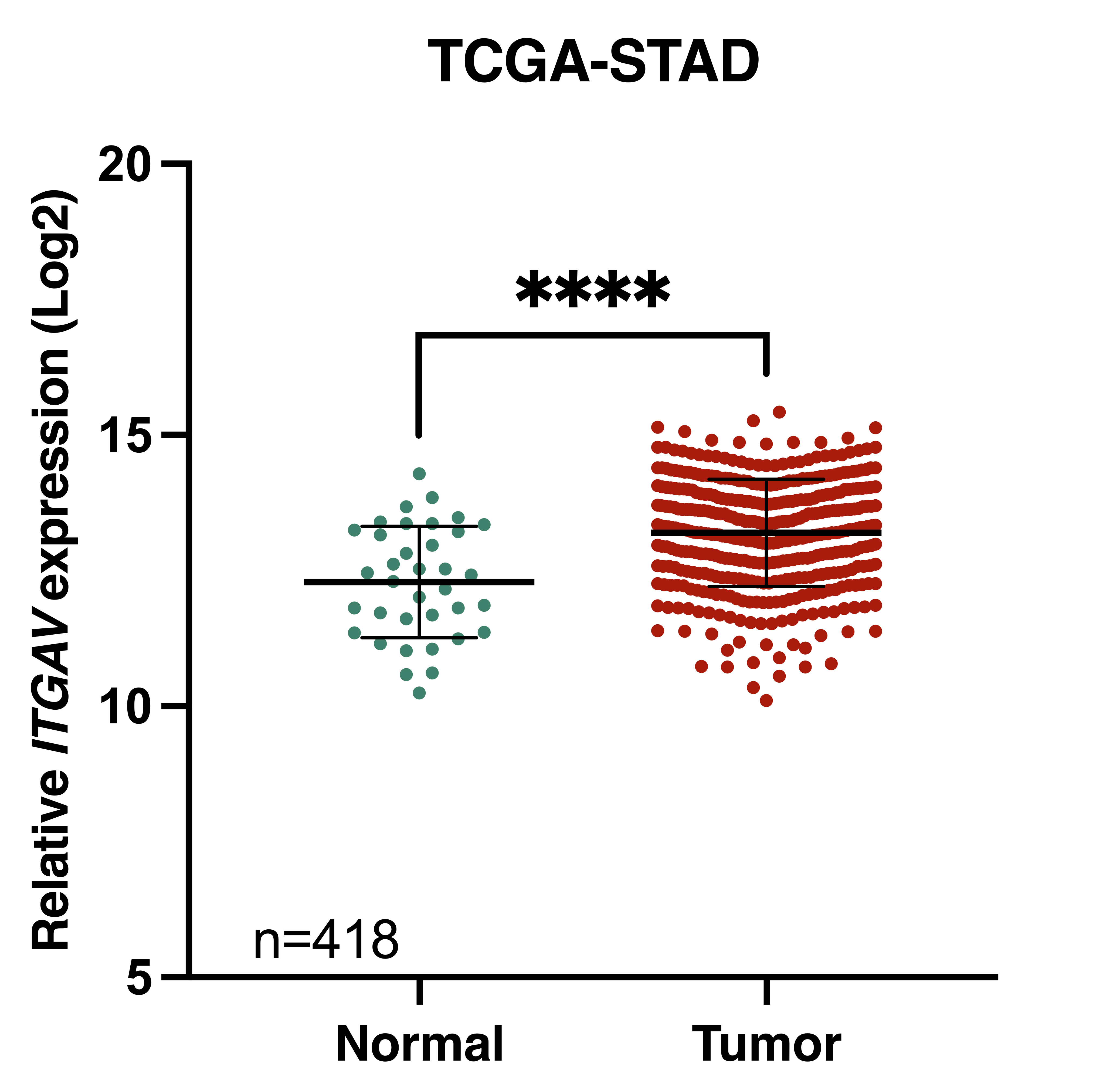

Supplement: Supplementary file 1 [file curroncol-31-00099-s001.zip › supplemental figures/Figure S1/STAD.tiff]

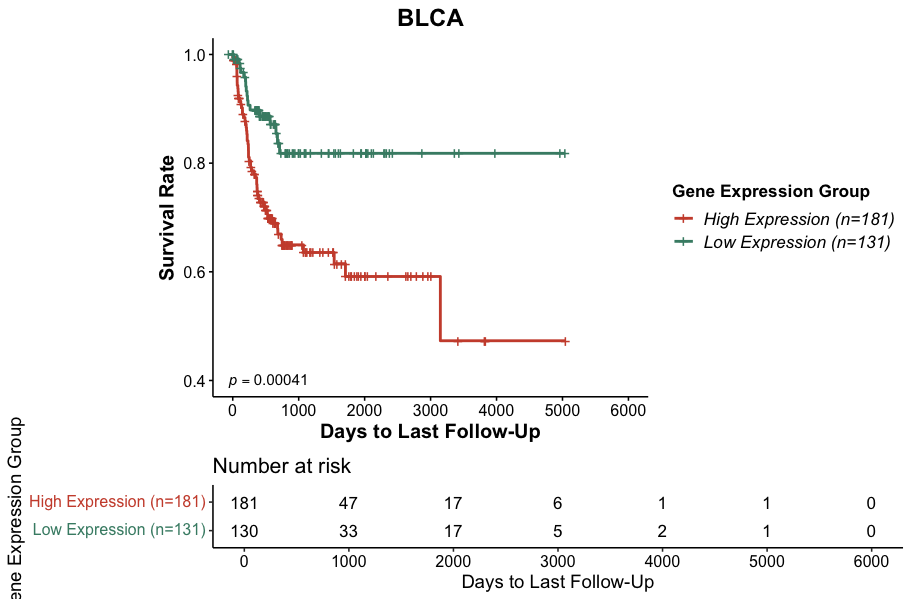

Supplement: Supplementary file 1 [file curroncol-31-00099-s001.zip › supplemental figures/Figure S2/BLCA p.tiff]

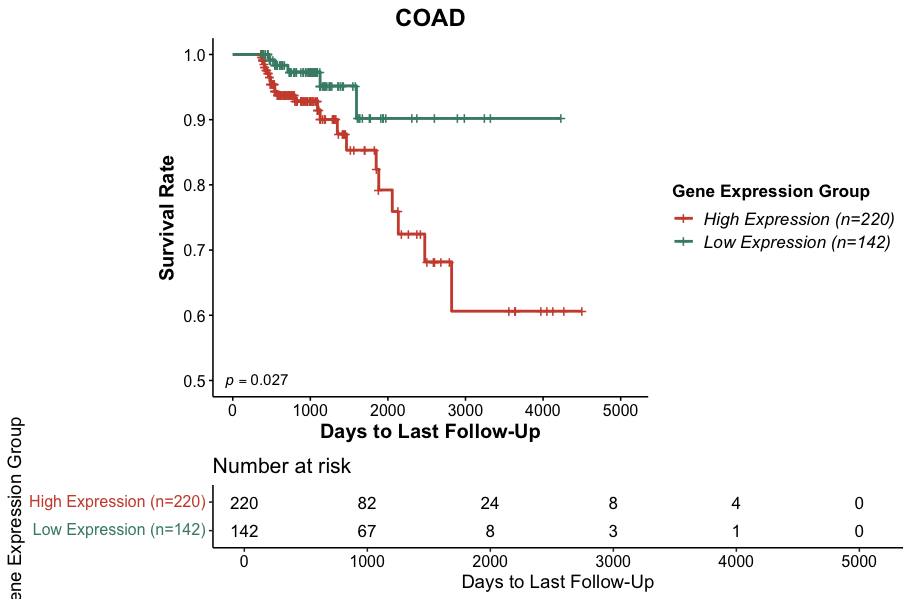

Supplement: Supplementary file 1 [file curroncol-31-00099-s001.zip › supplemental figures/Figure S2/COAD p.tiff]

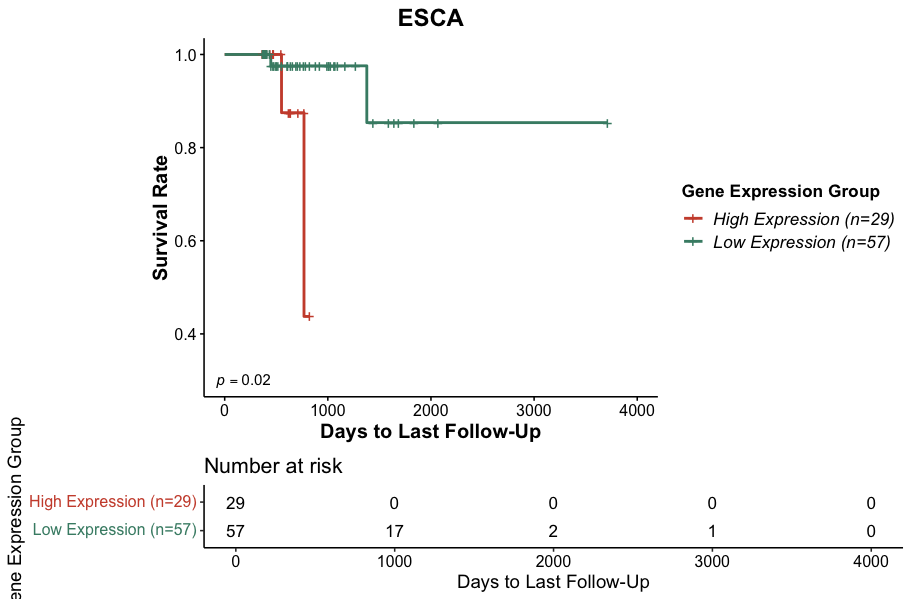

Supplement: Supplementary file 1 [file curroncol-31-00099-s001.zip › supplemental figures/Figure S2/ESCA p.tiff]

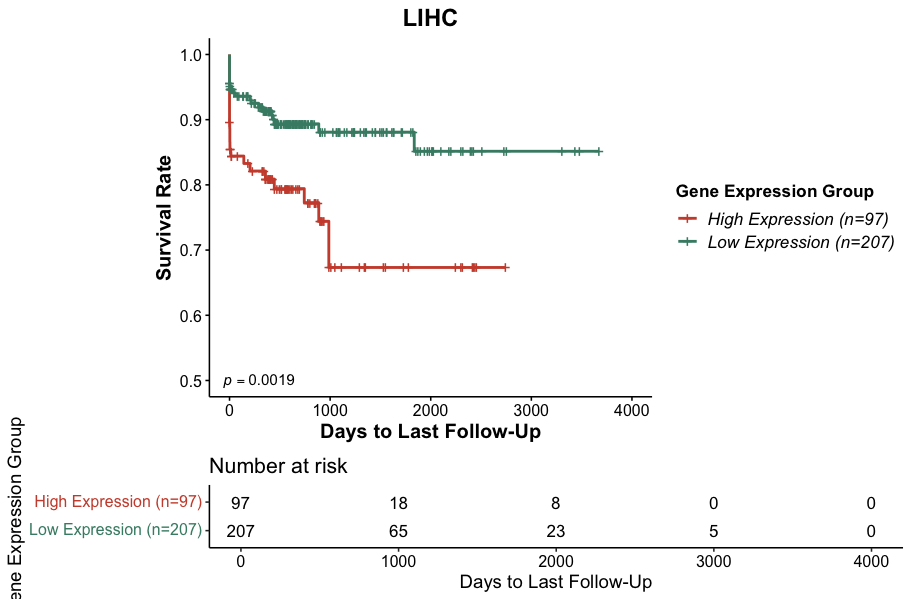

Supplement: Supplementary file 1 [file curroncol-31-00099-s001.zip › supplemental figures/Figure S2/LIHC p.tiff]

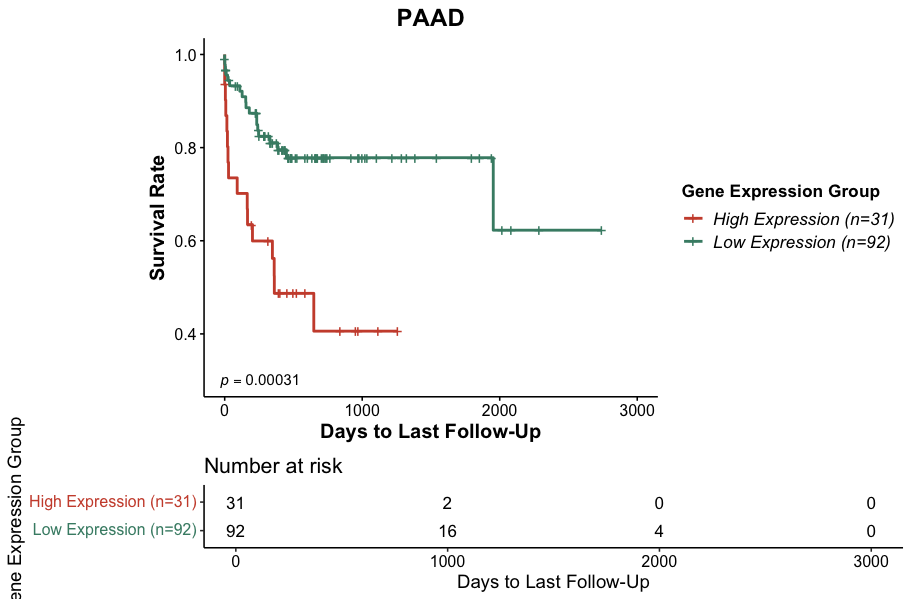

Supplement: Supplementary file 1 [file curroncol-31-00099-s001.zip › supplemental figures/Figure S2/PAAD p.tiff]

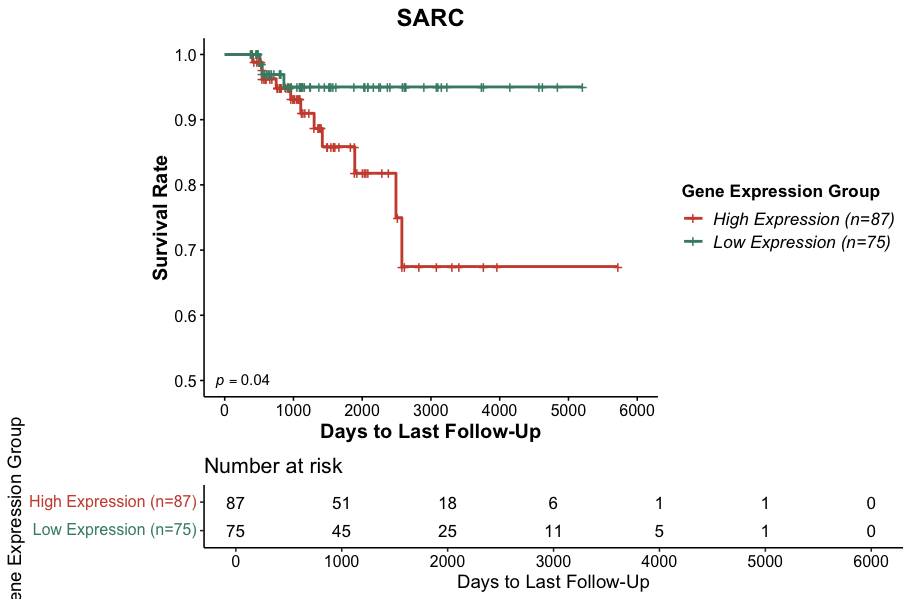

Supplement: Supplementary file 1 [file curroncol-31-00099-s001.zip › supplemental figures/Figure S2/SARC p.tiff]

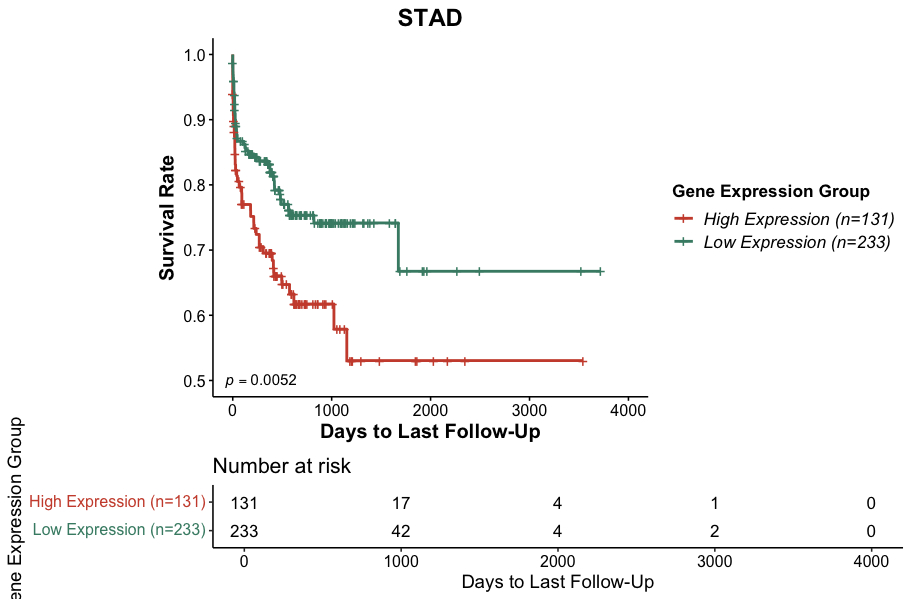

Supplement: Supplementary file 1 [file curroncol-31-00099-s001.zip › supplemental figures/Figure S2/STAD p.tiff]
